# Role of Tuberculosis in the Etiology of Myeloid Leukemia

**Published:** 1913-09

**Authors:** 


Role of Tuberculosis in the Etiology of Myeloid Leukemia. A. Nanta. (Arch. des Mal. du Coeur, des Vaisseaux, et du Sang, 1913, VI, 38.) As the result of the examination of cases reported in the literature and of the study of a case occuring in the hospital service of Dr. Rispal the author concludes (1) that attenuated tuberculosis of the lymphnodes is able to determine a diffuse myeloid hyperlasia which reproduces the syndrome of myeloid leukemia. (2) That in the majority of cases the relation between the two diseases is brought out by the demonstration at autopsy of an old latent tuberculosis; in some cases, furthermore, by the development of granulomata (granulie) which complicate the leukemia and which attack the hematopoitic organs almost exclusively. (3) That the myeloid reaction becomes less and even disappears before the acuity of the infection. (4) That combined lesions of tuberculosis and leukemia may present, whatever may be its significance, a new histologic form, which is devoid, with the exception of the presence of the tubercle bacillus, of the usual histrologic picture: foci of necrosis surrounded by zones of myeloid proliferation and containing bacilli. This form is sometimes quite like lymphogranuloma. The author thinks that tuberculosis is able to start up a myeloid reaction accompanied with myelemia, which may be extensive and permanent and of which the symptoms may be very pronounced. In such cases myeloid leukemia should be considered as a syndrome and not as an iodopathic disease.

